# Bu-Zhong-Yi-Qi Granule Enhances Colonic Tight Junction Integrity via TLR4/NF-*κ*B/MLCK Signaling Pathway in Ulcerative Colitis Rats

**DOI:** 10.1155/2021/6657141

**Published:** 2021-03-09

**Authors:** Xiuhong Kang, Mengdi Jia, Luqing Zhao, Shengsheng Zhang

**Affiliations:** Digestive Disease Center, Beijing Hospital of Traditional Chinese Medicine, Capital Medical University, Beijing, China

## Abstract

**Background:**

Bu-zhong-yi-qi granule (BZYQ), a sort of Chinese herbal medicine, has exhibited therapeutic effects on ulcerative colitis (UC). However, the mechanism of BZYQ has not been fully clarified. This study was aimed at investigating the effects of BZYQ on UC rats model and at exploring its potential mechanism.

**Methods:**

The UC rats were established by enema of trinitrobenzene sulfonic acid (TNBS). The therapeutic effects of BZYQ treatment were evaluated by disease activity index (DAI), colon macroscopic damage index (CMDI) scores, and histological observation. The mRNA levels of tumor necrosis factor-*α* (TNF-*α*), interleukin-1*β* (IL-1*β*), and interleukin-10 (IL-10) were measured by quantitative real time-polymerase chain reaction (qPCR). The expression of tight junction (TJ) proteins, occludin and claudin-1, in the colon was determined by Western blot and immunofluorescence. The expression of toll-like receptors 4 (TLR4), nuclear factor-kappa B (NF-*κ*B), p-NF-*κ*B, myosin light chain kinase (MLCK), MLC, and p-MLC levels in colon was determined by Western blot or qPCR.

**Results:**

The results showed that BZYQ could attenuate DAI, CMDI, and histological inflammation. TJ proteins expression was decreased in UC rats, but treatment with BZYQ restored the expression of occludin and claudin-1. In addition, BZYQ administration ameliorated UC-associated increase in the production of TNF-*α*, IL-1*β*, and the expression of TLR4, NF-*κ*B, p-NF-*κ*B, MLCK, MLC, and p-MLC, while BZYQ administration increased the production of IL-10.

**Conclusions:**

The therapeutic effect of BZYQ on UC is at least partially through regulation of the secretion of some inflammatory cytokines and improvement of TJ integrity via TLR4/NF-*κ*B/MLCK pathway.

## 1. Introduction

Ulcerative colitis is a chronic idiopathic inflammatory bowel disease characterized by persistent mucosal inflammation beginning in the rectum and extending to the proximal end. Typical presenting symptoms include intermittent bloody diarrhea, abdominal pain, tenesmus, and others [1]. In the past two decades, there has been an increasing tendency in incidence and prevalence of UC [2].

Multiple pathogeneses have been demonstrated in occurrence and development of UC; intestinal barrier is a pivotal pathogenic factor [3, 4]. Barrier dysfunction is the main driver of ulcerative colitis. This view is supported by the fact that there is a decrease in goblet cells and a decrease in the permeability of the mucous barrier in patients with active ulcerative colitis [5]. Recent experimental evidence indeed implicates a crucial function of barrier dysfunction in the onset of inflammatory bowel disease [6]. IBD can be caused by the dysfunction of intestinal epithelial cell, which is the internal molecular circuit to control the homeostasis, renewal, and repair of intestinal epithelial cells [7]. As a crucial structural factor of this barrier, tight junction (TJ) proteins can maintain intestinal mucosal barrier, prevent invasion of bacterium and toxin, and thereby reduce the progression of UC [8]. Besides, TJ opening is driven by myosin light chain (MLC) phosphorylation, which depends on myosin light chain kinase (MLCK) activation [9]. Recent experimental evidence also implicates that MLCK leads to a mechanical tension-generated opening of the TJ barrier by contracting the perijunctional actomyosin filaments [10, 11]. Experiments have proved that increasing TJ permeability was mediated by toll-like receptor 4 (TLR4)/MyD88 signal-transduction pathway upregulation of MLCK expression [11]. TLR4 (toll-like receptor 4) is a key pattern-recognition receptor for commensal recognition in gut innate immunity and it is overexpressed on the surface of inflamed colon [12, 13].

Today, the pharmacologic management of UC mainly relies on 5-aminosalicylates, corticosteroids, and immunosuppressants and biologic therapies [14], but the side effects of these treatment still exist [15, 16]. In recent years, as an alternative treatment modality for the treatment of UC, traditional Chinese medicine (TCM) has demonstrated observably clinical efficacy [17]. TCM treatment of ulcerative colitis is based on syndrome differentiation, and TCM believes that ulcerative colitis has the problem of spleen deficiency. Bu-zhong-yi-qi granule (BZYQ) is a classical prescription for curing spleen deficiency in traditional Chinese medicine, which has been used for a long time in clinic, and it is often used in the treatment of ulcerative colitis in China. It is composed of 10 ingredients, including Astragalus, Dangshen, Liquorice, *Atractylodes macrocephala*, Tangerine peel, Cohosh, Bupleurum, Angelica, Ginger, and Jujube. Studies have shown that BZYQ treatment has a protective effect on 5-FU-induced intestinal mucositis in mice, and concluded that BZYQ treatment may inhibit the upregulation of inflammatory cytokines [18], and experiments also have proved BZYQ could ameliorate patients with chronic nephritis through TLRs/MyD88 signaling pathway [19]. Active components of BZYQ, such as Astragalus polysaccharides, have been implicated to protect against DSS-induced colitis by inhibiting NF-*κ*В activation [20]. However, the molecular mechanism by which BZYQ inhibits ulcerative colitis is unclear. In this study, we aim to study the therapeutic efficacy of BZYQ treatment on UC. In addition, we analyzed the expression of tight junction-related proteins to study the protective mechanism of BZYQ's mucosal barrier.

## 2. Materials and Methods

### 2.1. Experimental Materials

#### 2.1.1. Animals

This animal experiment complies with the ARRIVE guidelines and the National Institutes of Health Guide for the Care and Use of Laboratory Animals (NIH Publications No. 8023, revised 1978). Specific pathogen-free male Wistar Rats (250 g ± 10 g) were acquired from the Chinese People's Liberation Army Military Medical Academy Experimental Animal Center. Rats were housed in the Institute of Basic Theory, China Academy of Chinese Medical Sciences (The Approval Number is 2017-098). All rats were kept in a standardized environment at a 12-hour light/dark cycle (lights on at 8 : 00 a.m.) with a normative temperature (21–23°C) and humidity (50% ± 5%). All the rats had free access to food and water. The study was approved by the Animal Care and Use Committee of China Academy of Traditional Chinese Medicine.

#### 2.1.2. Induction of UC in Rats

Intracolonic injection with TNBS which induces UC in rats is an acknowledged modeling method [21]. Briefly, rats were fasted for 36 hours but allowed free access to water. The rats were deeply anesthetized by inhaling Ulan (epin pharmaceutical, c002160902); then, TNBS solution (sigma, P2297) was injected into the colon of the rats (100 mg/kg, dissolved in 50% ethanol according to the proportion of 1 : 1) by inserting a 8 cm medical-grade tube into the anus. After injection of the TNBS, the rats were kept in a head-down position for 30 min to prevent leakage [22].

#### 2.1.3. Drugs and Administration

They were as follows: Bu-zhong-yi-qi granule (BZYQ) (batch number: Z20040120, Beijing Han Dian Pharmaceutical Co., Ltd., Beijing, China); Sulfasalazine Enteric-coated Tablets (SASP) (batch number: H31020557, Shanghai SINE Pharmaceutical Co., Ltd, Shanghai, China). According to the equivalent dose-ratio table of human and animal body surface area, the dosage of the rats = X mg/kg × 70 kg × 0.018/0.2 kg (*X* is the adult clinical dosage) [23]. Twenty-four hours after induction of UC, treatment began and was continued for seven days.

The normal control (no handling) rats were classified as control group; UC rats were divided into three groups: model group, the positive control group: SASP group (0.315 g/kg), and BZYQ group (1.89 g/kg). After establishment of the model, the rats in positive control group and the BZYQ group were given intragastric administration with treatment, respectively; the control group and model group were given distilled water. All groups were treated for 7 consecutive days. After being administered by gastric gavage for 7 days, all rats were killed with the method of cervical dislocation and the colon was excised for the following experiments.

#### 2.1.4. Ultra Performance Liquid Chromatography-Tandem Mass Spectrometry (UPLC-MS/MS) Analysis

Chromatographic separation was performed by Thermo Scientific u3000 fast liquid chromatography. A Rapid Separation LC spectrometer and hybrid quadrupole Orbitrap mass spectrometer were employed. The voltage of positive and negative ion source was 3.7 kv and 3.5 kv, respectively. The capillary heating temperature was 320°C. The warping pressure was 30 psi and the auxiliary gas pressure was 10 psi. Volume heating evaporation temperature was 300°C. The warping gas and auxiliary gas were both nitrogen. The collision gas was nitrogen and the pressure was 1.5 mtorr. The liquid quality system was controlled by Xcalibur 2.2 SP1.48 software. Data acquisition and targeted metabolite quantitative processing were operated by the software.

### 2.2. DAI, CMDI, and Histological Evaluation

#### 2.2.1. Disease Activity Index (DAI)

A disease activity index (DAI) score was used for the evaluation of the severity of colonic inflammation in rats after 7 days of the treatment: DAI  = (score of weight loss + stool consistency + degree of hematochezia)/3 [24]. The details are shown in Table 1.

#### 2.2.2. Colon Macroscopic Damage Index (CMDI)

After 7 days of the treatment, the distal colon (8 cm from the anus) was immediately obtained and longitudinally incised from mesentery. Then, the opened sample of colon was washed out thoroughly by cold normal saline and measured by blind evaluation via colon macroscopic damage index (CMDI) involving severity of inflammation and existence of ulcer [25–27]. The scoring criteria were described as follows: 0: no ulcer, no inflammation; 1: no ulcer, local hyperemia; 2: ulcer without hyperemia; 3: ulcer and inflammation at one site only; 4: two or more sites of ulcer and inflammation; and 5: ulcer extending more than 2 cm.

#### 2.2.3. Histological Evaluation

Four samples of rat for each group were selected by random choice, and then the samples of colonic tissue from each rat were fixed in 4% buffered formaldehyde and embedded in paraffin. Sections were sliced to a thickness of 5 *μ*m and stained with hematoxylin-eosin. The sections were detected under microscope (NanoZoomer S60, Japanese) by a researcher unaware of the treatment.

### 2.3. Quantitative Real-Time Polymerase Chain Reaction (qPCR)

The total RNA was extracted from colon samples with Trizol reagent (Thermo Fisher Scientific, USA) and 5 ug of total RNA was reversely transcribed to cDNA according to the reagent instructions. The expression levels of TLR4, MLCK, NF-*κ* B, TNF-*α*, IL-1 *β*, and IL-10 were detected by SYBR Master Mix (Promega, USA) and the CFX 96 Real-Time PCR System (BIO-RAD, USA). RT-PCR was based on our previous report. The primers are shown in Table 2.

### 2.4. Western Blot

The colonic tissue was homogenized by applying ice-cold RIPA buffer containing 1 mM PMSF (Solarbio, China). The protein concentration was assessed by BCA assay. The equal amount of protein (80 *μ*g) of each sample was separated by sodium dodecyl sulfate-polyacrylamide gel electrophoresis (SDS-PAGE) using 10% polyacrylamide gels and electrophoretically transferred to polyvinylidene fluoride membranes after separation. The membrane was then incubated with the following primary antibodies: anti-occludin (1 : 400, Santa CruzBiotechnology), anti-claudin-1 (1 : 1500, Abcam) and anti-*β*-actin (1 : 1500, ZSGB-BIO), anti- NF-*κ*B (1 : 1000,YM3111, Immunoway, USA), anti-p- NF-*κ*B (1:1500,YP0193, Immunoway, USA), anti-TLR4 (1 : 1000 dilution, Proteintech), anti-MLC (1:400,Abcam), and anti-p-MLC (1:400, CSL) at 4°C overnight. Blots were washed three times by TBST (Solarbio, China); then, the membrane was incubated with matching secondary antibodies: DyLight 680-labeled goat anti-rabbit or goat anti-mouse (both 1 : 10000, KPL). The specific protein bands on the membrane were visualized and the protein levels were analyzed with Image-ProPlus.

### 2.5. Immunofluorescence

The colonic sections were fixed in OCT and then sliced at 6 *μ*m thickness by freezing microtome (ThermoFisher, USA). Sections were incubated with anti-occludin (1 : 50, Santa Cruz Biotechnology) or anti-claudin-1 (1 : 40, Abcam) at 4°C overnight. After three times repeated TNBS washing, and then followed by incubation with rabbit anti-goat or goat anti-rabbit (1 : 100, ZSGB-BIO) 2nd antibody for 30 min at room temperature, respectively, the sections were mounted with DAPI (ZSGB-BIO) and detected by fluorescence microscope (Pannoramic MIDI, USA).

### 2.6. Statistical Methods

The statistical analyses were examined by applying SPSS 17.0 software (SPSS, Chicago, IL, USA) and each value was expressed as means ± SE. All the original data in the study were distributed normally and conformed to homogeneity of variance. The differences among the different groups were analyzed using one-way analysis of variance (ANOVA) followed by least-significant difference (LSD) test to compare the differences between every two groups. *P* < 0.05 was considered statistically significant.

## 3. Results

### 3.1. Identification of Major Components of BZYQ Granule

**Figure 1:**
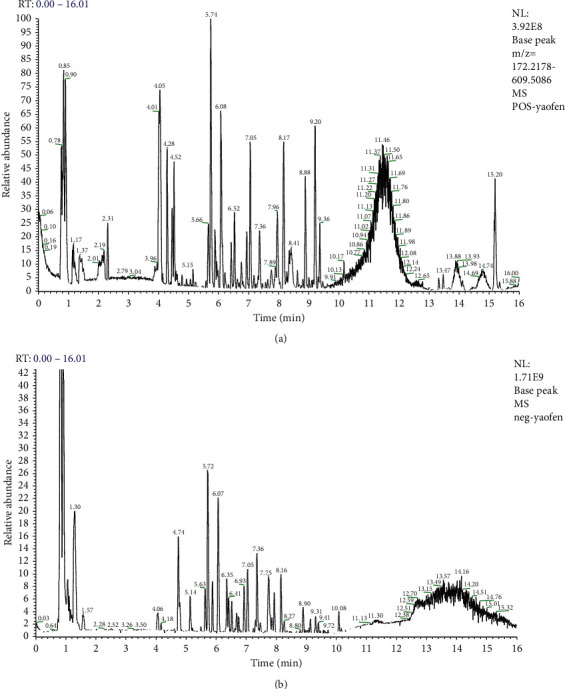
Identification of major components of BZYQ granule examined by UPLC-MS/MS. Data were collected and proceeded by software MassLynx 4.1. The positive (a) and negative (b) ion chromatograms of BZYQ granule are shown as indicated.

To identity the main ingredients, samples of BZYQ granule were evaluated by UPLC-MS/MS. The total positive (Figure 1(a)) and negative (Figure 1(b)) ion chromatograms of BZYQ granule demonstrated the composition of all ingredients (Figure 1(a)).

### 3.2. BZYQ Treatment Alleviated TNBS-Induced Colitis

**Figure 2:**
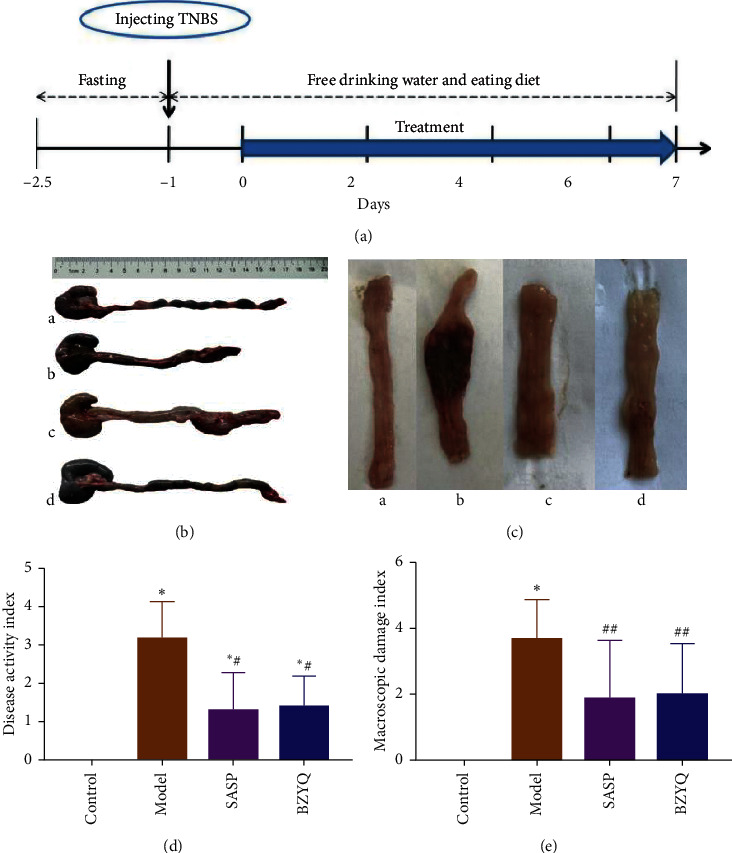
(a) Induction of UC in rats and the treatment protocol. (b, c) Length and gross appearances of colon in four groups. (b) A: control group; B: model group; C: SASP group; D: BZYQ group. (c) A: control group: showing no inflammation; B: model group, showing marked hyperemia, inflammation, and large ulcer; C: SASP group, showing reduced hyperemia and inflammation; D: BZYQ group: showing reduced hyperemia and inflammation. (d, e) Concentrations of DAI and CMDI of each group.^*∗*^*P* < 0.05 compared with control group; ^#^*P* < 0.05, ^##^*P* < 0.01 compared with model group (means ± SE, *n* = 6).

Firstly, we observed the clinical features, weight loss, and loose stool with mucus and/or blood in model group. In SASP and BZYQ on the 7th day, bloody stools were alleviated, characteristics of stools were better formed, and weight loss was relieved. The length and gross appearance of colon were evaluated as shown in Figures 2(b) and 2(c); the colon from model group showed obvious edema, hyperemia, inflammation, and ulcer. After treatment with SASP or BZYQ, the above damaged signs of colon were significantly alleviated.

DAI can reflect the severity of UC through three aspects, which include weight loss, stool consistency, and hematochezia. CMDI is known to be a macroscopic evaluation of inflammation. As shown in Table 3, compared with the control rats, UC rats showed a significant increase in DAI and CMDI (3.17 ± 0.39 vs 0.00 ± 0.00, *P* < 0.01; 3.67 ± 0.49 vs 0.00 ± 0.00, *P* < 0.01). After the treatment, DAI and CMDI in SASP group and BZYQ group were reduced more than those in the model group (1.29 ± 0.37 or 1.38 ± 0.30 vs 3.17 ± 0.39, *P* < 0.01; 1.86 ± 0.67 or 2.00 ± 0.58 vs 3.67 ± 0.49, *P* < 0.05). And there were no statistically significant differences between the two groups (*P* > 0.05) (Figures 2(d) and 2(f)).

### 3.3. BZYQ Treatment Ameliorated the Histopathological Responses

**Figure 3:**
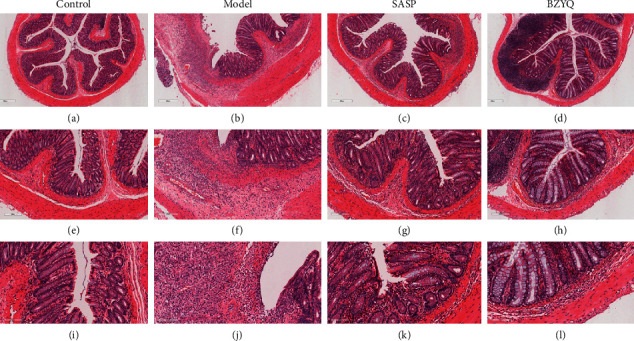
The morphologic changes of colon in four groups. (a, e, and i) Control group: showing normal colonic mucosa (H&E staining, 4 ×, 10 ×, 20 ×). (b, f, and j) Model group: showing massive ulcers, changes in crypt structure and loss of inflammation (H&E staining, 4 ×, 10 ×, 20 ×). (c, g, and k) SASP group: showing marked reduction of inflammation and nearly intact mucosa (H&E staining, 4 ×, 10 ×, 20 ×). (d, h, and l) BZYQ group: showing marked reduction of inflammation and nearly intact mucosa (H&E staining, 4 ×, 10 ×, 20 ×).

As shown in Figure 3, intestinal mucosa had integrity, structure was clear, and no inflammation was observed in control group, whereas intestinal mucosa was incomplete, and extensive infiltration of inflammation and massive ulceration were found in model group. In each treatment group, intestinal mucosa was relatively complete, and ulcer healing and small amount of inflammation were observed.

### 3.4. BZYQ Treatment Regulated the Expression of Inflammatory Cytokines in Ulcerative Colitis Rats

**Figure 4:**
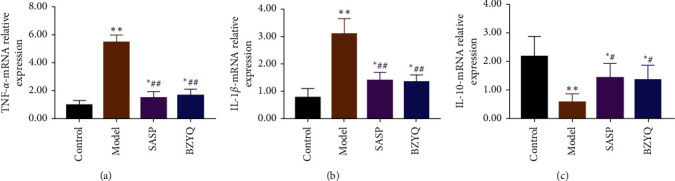
Concentrations of TNF-*α* (a), IL-1*β* (b), and IL-10 (c) of colon in each group.^*∗*^*P* < 0.05, ^*∗∗*^*P* < 0.01 compared with control group; ^#^*P* < 0.05, ^##^*P* < 0.01 compared with model group (means ± SE, *n* = 6).

TNF-*α* and IL-1*β* levels were significantly higher in the colon of UC rats than in the control group (TNF-*α*: 5.47 ± 0.21 vs 0.98 ± 0.13, *P* < 0.01; 1L-1*β*: 3.10 ± 0.23 vs 0.77 ± 0.13, *P* < 0.01). IL-10 level was significantly lower in the colon of UC rats than in the control group (0.55 ± 0.13 vs 2.17 ± 0.29, *P* < 0.01). More importantly, BZYQ treatment significantly reduced TNF-*α* and IL-1*β* levels and enhanced IL-10 level in UC rats (TNF-*α*: 1.64 ± 0.19 vs 5.47 ± 0.21, *P* < 0.01; IL-1*β*: 1.35 ± 0.11 vs 3.10 ± 0.23, *P* < 0.01; IL-10 : 1.34 ± 0.21 vs 0.55 ± 0.13, *P* < 0.01) (Table 4). Furthermore, there was no significant difference in inflammatory cytokines levels between the SASP group and the BZYQ group (Figure 4).

### 3.5. BZYQ Treatment Increased the Expression of TJ Proteins in UC Rats

**Figure 5:**
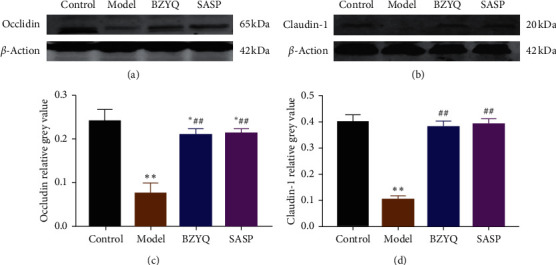
Effect of BZYQ treatment on expression of TJ proteins of colon in each group. (a, b) Western blot bands of occludin and claudin-1; (c, d) the expression level was normalized to the house keeping protein *β*-actin. ^*∗*^*P* < 0.05, ^*∗∗*^*P* < 0.01 compared with control group; ^#^*P* < 0.05, ^##^*P* < 0.01 compared with model group (means ± SE, *n* = 6).

**Figure 6:**
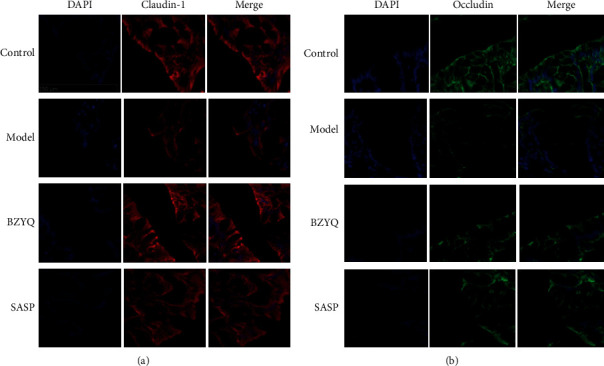
Representative photomicrographs of immunofluorescence of occludin (a) and claudin-1 (b). These pictures show a decrease in the expression of both occludin and claudin-1 in the model group and BZYQ treatment could restore the expression of two TJ proteins.

TJ proteins as important components of mechanical barrier protect the mucosa from invasion of bacterial endotoxin and other toxin [28]. We tested whether BZYQ treatment restores epithelial barrier by regulating the expression of TJ proteins. Owing to the necessary roles of occludin and claudin-1 in sustaining a tight barrier, their expressions were detected by Western blot in the present study. The expression levels of occludin and claudin-1 were significantly reduced in the UC rats compared with the control group (occludin: 0.07 ± 0.01 vs 0.24 ± 0.01, *P* < 0.01; claudin-1: 0.10 ± 0.01 vs 0.40 ± 0.01, *P* < 0.01) (Figure 5). Meanwhile, the expressions of both occludin (Figure 6(a)) and claudin-1(Figure 6(b)) were significantly decreased in UC rats by immunofluorescence staining. After treatment of BZYQ, both Western blot and immunofluorescence staining showed that the expressions of occludin and claudin-1 were significantly improved in UC rats. The above results demonstrated that it plays pivotal role in restoring epithelial barrier function that BZYQ treatment increases the expression of TJ proteins.

### 3.6. BZYQ Treatment Modulated TLR4/NF-*κ*B/MLCK Signaling

**Figure 7:**
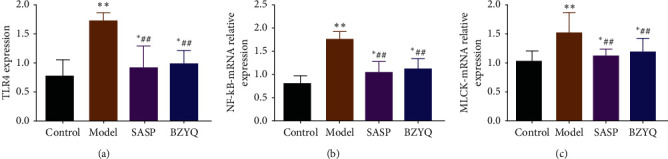
Concentrations of TLR4 (a), NF-*κ*B (b), and MLCK (c) of colon in each group.^*∗*^*P* < 0.05, ^*∗∗*^*P* < 0.01 compared with control group; ^#^*P* < 0.05, ^##^*P* < 0.01 compared with model group (means ± SE, *n* = 6).

To further evaluate the effects of BZYQ treatment, we investigated the role of TLR4, NF-*κ*B, p-NF-*κ*B, MLCK, MLC, and p-MLC in UC inflammation and tight junction integrity. In this study, we attempted to determine whether TLR4 related pathway is involved in treatment of BZYQ for UC [29–31]. As shown in Figure 7, the mRNA levels of TLR4, NF-*κ*B, and MLCK were significantly increased in the colon of UC rats compared with the control group (TLR4: 1.70 ± 0.15 vs 0.54 ± 0.09, *P* < 0.01; NF-*κ*B: 1.77 ± 0.06 vs 0.80 ± 0.07, *P* < 0.01; MLCK:1.51 ± 0.15 vs 1.03 ± 0.07, *P* < 0.01). More importantly, UC rats showed a much lower level of TLR4, NF-*κ*B, and MLCK after BZYQ treatment (TLR4: 0.99 ± 0.11 vs 1.70 ± 0.15, *P* < 0.01; NF-*κ*B: 1.11 ± 0.10 vs 1.77 ± 0.06, *P* < 0.05; MLCK: 1.19 ± 0.09 vs 1.51 ± 0.15, *P* < 0.05). In addition, there was no significant difference between the BZYQ group and the SASP group in TLR4, NF-*κ*B, and MLCK (*P* > 0.05).

**Figure 8:**
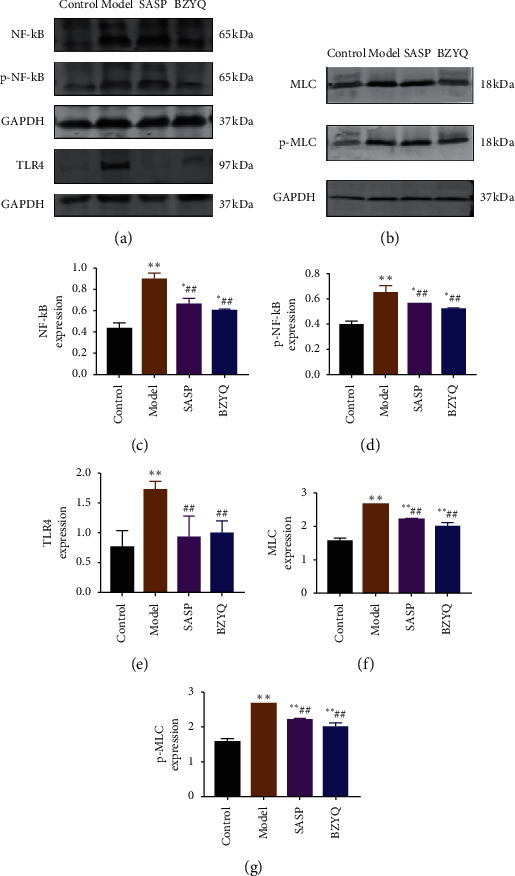
Effects of BZYQ treatment on NF-*κ*B, p-NF-*κ*B, TLR4, MLC, and p-MLC expression in UC mouse. (a) Western blot analysis of NF-*κ*B, p-NF-*κ*B3, and TLR4 protein levels in each group. (b) Western blot analysis of MLC and p-MLC protein levels in each group. (c, d, and e) Quantitative analysis of NF-*κ*B, p-NF-*κ*B, and TLR4. Expression was standardized to GAPDH expression. Data are shown as the mean ± SE (*n* = 3 rats per group). ^*∗*^*P* < 0.05, ^*∗∗*^*P* < 0.01 compared with control group; ^#^*P* < 0.05, ^##^*P* < 0.01 compared with model group. (f and g) Quantitative analysis of MLC and p-MLC. Expression was standardized to GAPDH expression. Data are shown as the mean ± SE (*n* = 3 rats per group). ^*∗*^*P* < 0.05, ^*∗∗*^*P* < 0.01 compared with control group; ^#^*P* < 0.05, ^##^*P* < 0.01 compared with model group.

As reported in the literature, NF-*κ*B is highly expressed in UC, and TLR4 is involved in activation of NF-*κ*B signaling. In addition, TLR4-NF-*κ*B-MLCK is a classical signaling pathway and it has been demonstrated to modulate inflammatory response. As shown in Figures 8(a), 8(c), and 8(d), protein expression levels of NF-*κ*B and p-NF-*κ*B were upregulated in TNBS-induced rats compared to the control group, and the level of TLR4 was markedly increased (^*∗∗*^*P* < 0.01, Figures 8(a), 8(c)–8(e)). Myosin light chain kinase (MLCK) plays an indispensable role in the activation of actin and myosin filament contraction, and it has been confirmed that epithelial TJ opening is driven by myosin light chain (MLC) phosphorylation [9]. As shown in Figure 8(b), 8(f), and 8(g), protein expression levels of MLC and p-MLC were upregulated in TNBS-induced rats compared to the control group (^*∗∗*^*P* < 0.01, Figure 8(b), 8(f), and 8(g)). Levels of TLR4, NF-*κ*B, p-NF-*κ*B, MLC, and p-MLC were decreased after treatment of BZYQ (^##^*P* < 0.01).

## 4. Discussion

Defective intestinal epithelial TJ barrier is an important pathogenic factor. It promotes the development of intestinal inflammation by increasing the intestinal permeability of intestinal derived bacterial antigens. This study was designed to test the hypothesis that BZYQ treatment could exert its effect against UC through anti-inflammation and improved barrier. Our present study proved that BZYQ was effective in treating UC in rats, and the underlying therapeutic mechanisms of BZYQ, maybe the regulation of the TLR4 pathway, suppressed MLCK expression and the activity of NF-*κ*B, while there was a subsequent increase in the expression levels of occludin and claudin-1.

As a classic patent drug of TCM, BZYQ granule plays a role of promoting Yang and relieves diarrhea [32]. BZYQ consists of Astragalus, Dangshen, Liquorice, *Atractylodes macrocephala*, Tangerine peel, Cohosh, Bupleurum, Angelica Ginger, and Jujube. Modern pharmacological studies indicate that the chemical constituents of BZYQ are comprised of ammonium glycyrrhetate, saikosaponin A, hesperidin, and ferulic acid, which collectively play a significant role in ameliorating clinical symptoms [33, 34]. The active component Astragalus polysaccharides have been implicated to protect against dextran sulfate sodium-induced colitis by inhibiting NF-*κ*В activation [19]. However, the mechanism of BZYQ in the treatment of UC is not been fully clarified. In order to explore the mechanism of BZYQ in vivo, we constructed TNBS-induced ulcerative colitis model rats and used SASP as a positive drug, which has been used as the standard treatment of UC [35]. BZYQ treatment significantly decreased DAI and CMDI scores which are correlated with the severity of UC (Figure 2). Furthermore, the protective effect of high and middle dose BZYQ was comparable to that of SASP. In histological examination, similar results were found: BZYQ treatment could significantly restore the structure of intestinal mucosa and reduce inflammatory infiltration (Figure 3).

Many studies have demonstrated that the levels of proinflammatory cytokines, such as interleukin IL-1*β* and TNF-*α*, are raised in IBD and play a central part in inflammatory response [36–38]. In addition, IL-10, as a protective factor, can inhibit the proinflammatory response and help maintain the integrity and homeostasis of epithelial tissue layers [39]. Our study has shown that BZYQ mitigated rat colonic inflammation by decreasing colonic production of proinflammatory cytokine TNF-*α* and IL-1*β*, while increasing that of anti-inflammatory cytokine IL-10 (Figure 4). The above results have proved that BZYQ and SASP may have similar therapeutic efficacy in the treatment of UC rats induced by TNBS.

**Figure 9:**
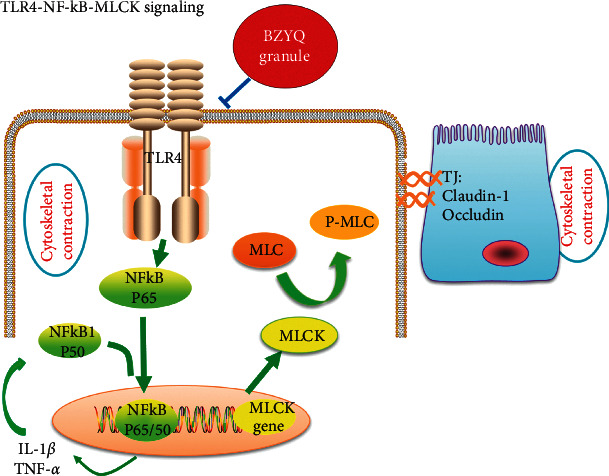
TNBS-induced activation of the toll-like receptor (TLR) 4 signal transduction signaling cascade, which leads to the activation of canonical NF-kB pathway. Canonical NF-*κ*B p65/p50 causes upregulation of myosin light chain kinases (MLCK) gene and protein expression, causing MLC phosphorylation, ultimately increasing tight junction (TJ) permeability in vivo. BZYQ treatment may inhibit activation of the toll-like receptor (TLR) 4 signal pathway to maintain intestinal epithelial function.

As we all know, intestinal barrier plays a key role in preventing harmful substances and pathogens from entering the submucosa via intestinal cavity. In intestinal permeability diseases, the defective intestinal tight junction (TJ) barrier allows intraluminal antigens to penetrate through the cell side and induces inflammatory reaction [40]. Evidence from clinical trials indicated that impaired intestinal barrier is connected with occurrence and development of UC [41, 42]. Occludin and claudin-1 as the major tight junction proteins constitute the basic structure of tight junctions in intestinal barrier [43–45]. Myosin light chain kinase (MLCK) is also a key effecter of barrier dysfunction and a potential therapeutic target [46]. MLCK has been implicated to be involved in TJ regulation during intestinal inflammation [47]. Myosin light chain kinase (MLCK) plays an indispensable role in the activation of actin and myosin filament contraction, and it has been confirmed that epithelial TJ opening is driven by myosin light chain (MLC) phosphorylation, which depends on the activation of MLCK [9]. In our present study, we found that BZYQ treatment significantly increased the expression of occludin and claudin-1 (Figures 5 and 6), while MLCK level was significantly decreased in BZYQ rats than in the UC rats (Figures 7 and 8), which may suggest that barrier integrity is involved in the curative mechanism of BZYQ for UC. BZYQ treatment ameliorated barrier integrity by increasing occludin and claudin-1 expression and decreasing MLCK expression in UC rats. Besides, in our present study, TLR4 and NF-*κ*B levels also significantly decreased in BZYQ rats compared to those in the UC rats (Figures 7 and 8). Primary intestinal epithelial cells (IEC) of normal mucosa TLR4 were barely detectable, while, in active IBD, the expression TLR4 was strongly upregulated in the intestinal epithelium [13]. TLR-mediated signaling pathway has been proved to be an ideal target for inflammatory diseases because of its regulation of inflammation and tissue integrity [48]. Several studies have evaluated the role of TLR4 in induction of epithelial barrier dysfunction and TLR4 has been studied in patients with chronic inflammatory bowel diseases [49, 50]. NF-*κ*B is an important downstream target of the TLR4 signal transduction pathway. TLR4/myeloid differentiation primary response (MyD) 88 pathway was a crucial upstream regulator of TAK-1 and NF-kB p50/p65 activation [51]. Our data showed BZYQ treatment causes low level of NF-*κ*B expression, which suggests that BZYQ treatment could inhibit NF-*κ*B activity. Furthermore, inhibition of MLCK can attenuate TNF-*α* induced nuclear translocation of p65 and phosphorylation of I*κ*B, which indicates MLCK plays a role in inducing activation of NF-*κ*B [52, 53]. As noted above, inhibiting key molecules of TLR4 pathway to improve tight junction proteins may be a mechanism in the treatment of BZYQ for UC (Figure 9).

## 5. Conclusion

In conclusion, we demonstrated that BZYQ treatment produced a therapeutic effect in UC rats and can reduce the expression of inflammatory factors. Furthermore, the underlying therapeutic mechanisms may be that BZYQ restores colonic barrier integrity via TLR4/NF-*κ*B/MLCK pathway. Due to the recurrence of traditional Chinese medicine, the effective ingredients need to be further studied, and other effective factors in the pathway also need to be further confirmed.

## Figures and Tables

**Table 1:** Disease activity index (DAI) score.

Score	Weight loss (%)	Stool consistency	Hematochezia
0	None	Normal	Negative
1	1-5		
2	5-10	Loose stool	Hemoccult positive
3	10-15		
4	>15	Liquid stool	Gross blood

**Table 2:** Primers used for RT-PCR.

Target gene	Primer sequence (5'-3')
*β*-Actin	Forward: 5´-AGAGGGAAATCGTGCGTGAC-3´
	Reverse: 5´-CCATACCCAGGAAGGAAGGCT-3´
TLR4	Forward: 5´-CCAGAGCCGTTGGTGTATCT-3´
	Reverse: 5´-TCAAGGCTTTTCCATCCAAC-3´
MLCK	Forward: 5´-GACGTGTTCACCCTGGTTCT-3´
	Reverse: 5´-TTTGTGCAGCATCAGTGACA-3´
NF-*κ*b	Forward: 5´-GGCAGCACTCCTTATCAA-3´
	Reverse: 5´-GGTG TCGTCCCATCGTAG-3´
TNF-*α*	Forward: 5´-AGAACTCCAGGCGGTGTCT-3´
	Reverse: 5´-TCCCTCAGGGGTGTCCTTAG-3´
IL-1*β*	Forward: 5´-ATAGCAGCTTTCGACAGTGAG-3´
	Reverse: 5´-GTCAACTATGTCCCGACCATT-3´
IL-10	Forward: 5´-GCAGGACTTTAAGGGTTACTTGG-3´
	Reverse: 5´-TCATTCTTCACCTGCTCCACTG-3´

**Table 3:** Effect of BZYQ treatment on the DAI and CMDI.

Group	N	DAI (means ± SE)	CMDI (means ± SE)
Control	7	0.00 ± 0.00	0.00 ± 0.00
Model	6	3.17 ± 0.39^∗∗^	3.67 ± 0.49^∗∗^
SASP	7	1.29 ± 0.37^∗∗##^	1.86 ± 0.67^∗#^
BZYQ	7	1.38 ± 0.30^∗∗##^	2.00 ± 0.58^∗#^

^*∗∗*^ *P* < 0.01, ^*∗*^*P* < 0.05 compared with control group;^##^*P* < 0.01, ^#^*P* < 0.05 compared with model group.

**Table 4:** Expression of the inflammatory cytokines.

Group	N	TNF-*α* (means ± SE)	IL-1*β* (means ± SE)	IL-10 (means ± SE)
Control	6	0.98 ± 0.13	0.77 ± 0.13	2.17 ± 0.29
Model	6	5.47 ± 0.21^∗∗^	3.10 ± 0.23^∗∗^	0.55 ± 0.13^∗∗^
SASP	6	1.51 ± 0.18^∗∗##^	1.39 ± 0.12^∗##^	1.42 ± 0.21^∗#^
BZYQ	6	1.64 ± 0.19^∗∗##^	1.35 ± 0.11^∗##^	1.34 ± 0.21^∗#^

^*∗∗*^ *P* < 0.01, ^*∗*^*P* < 0.05 compared with control group; ^##^*P* < 0.01, ^#^*P* < 0.05 compared with model group.

## Data Availability

The datasets used and analyzed during the current study are available from the corresponding author on reasonable request.

## Abbreviations

BZYQ: Bu-zhong-yi-qi granule
TNBS: Trinitrobenzene sulfonic acid
UC: Ulcerative colitis
SASP: Sulfasalazine enteric-coated tablets
DAI: Disease activity index
CMDI: Colon macroscopic damage index
TNF-*α*: Tumor necrosis factor-*α*
IL-1*β*: Interleukin-1 *β*
IL-10: Interleukin-10
TJ: Tight junction
TLR4: Toll-like receptor 4
NF-*κ*B: Nuclear factor-kappa B
MLCK: Myosin light chain kinase
TCM: Traditional Chinese medicine.
